# Determination and Quantification of Acetaldehyde, Acetone, and Methanol in Hand Sanitizers Using Headspace GC/MS: Effect of Storage Time and Temperature

**DOI:** 10.3390/ijerph21010074

**Published:** 2024-01-09

**Authors:** Ngoc Diem Kieu To, Jacob A. Theruvathu

**Affiliations:** Department of Natural Sciences, University of Houston-Downtown, Houston, TX 77002, USA; tok2@gator.uhd.edu

**Keywords:** hand sanitizer, alcohol, acetaldehyde, acetone, GC/MS, isotope, storage time, temperature

## Abstract

Accurate determination of the concentration of alcohols and their metabolites is important in forensics and in several life science areas. A new headspace gas chromatography–mass spectrometry method has been developed to quantify alcohols and their oxidative products using isotope-labeled internal standards. The limit of detection (LOD) of the analytes in the developed method was 0.211 µg/mL for methanol, 0.158 µg/mL for ethanol, 0.157 µg/mL for isopropanol, 0.010 µg/mL for n-propanol, 0.157 µg/mL for acetone, and 0.209 µg/mL for acetaldehyde. The precision and accuracy of the method were evaluated, and the relative standard deviation percentages were found to be less than 3%. This work demonstrates the application of this method, specifically in quantifying the concentration of oxidative products of alcohol and other minor alcohols found in hand sanitizers, which have become an essential household item since the COVID-19 pandemic. Apart from the major components, the minor alcohols found in hand sanitizers include methanol, isopropanol, and n-propanol. The concentration range of these minor alcohols found in ethanol-based hand sanitizer samples was as follows: methanol, 0.000921–0.0151 mg/mL; isopropanol, 0.454–13.8 mg/mL; and n-propanol, 0.00474–0.152 mg/mL. In ethanol-based hand sanitizers, a significant amount of acetaldehyde (0.00623–0.231 mg/mL) was observed as an oxidation product, while in the isopropanol-based hand sanitizer, acetone (0.697 mg/mL) was observed as an oxidation product. The concentration of acetaldehyde in ethanol-based hand sanitizers significantly increased with storage time and temperature, whereas no such increase in acetone concentration was observed in isopropanol-based hand sanitizers with storage time and temperature. In two of the selected hand sanitizers, the acetaldehyde levels increased by almost 200% within a week when stored at room temperature. Additionally, exposing the hand sanitizers to a temperature of 45 °C for 24 h resulted in a 100% increase in acetaldehyde concentration. On the contrary, the acetone level remained constant upon the change in storage time and temperature.

## 1. Introduction

The utilization of hand sanitizer products has become exceptionally prevalent worldwide throughout the coronavirus (COVID-19) pandemic time. Alcohol-based hand sanitizers are very effective and have been widely used for disinfecting microbes. The key mechanism in the disinfection process involves the denaturation of proteins and the disruption of the cell membrane of microbes by alcohol [1]. Among alcohols, ethanol and isopropanol have the highest antimicrobial activities, and hence they are the most common alcohols present in hand sanitizer products [2]. In addition to alcohol, other ingredients such as glycerol and hydrogen peroxide (H_2_O_2_) are also present in hand sanitizers [3,4]. To serve as a disinfectant, hand sanitizer should contain a certain concentration of alcohol. The typical concentration of alcohol in hand sanitizer ranges from 60–95%. According to the World Health Organization (WHO), formulations for hand hygiene products and ethanol-based products typically contain 80% alcohol, whereas isopropanol-based products contain 75% alcohol concentration [5]. Alcohol-based hand sanitizers are available in a variety of formats such as gel, liquid, and foam. In addition to alcohol-based hand sanitizers, alcohol-free hand sanitizers are also effective in disinfecting microbes [6,7]. Benzalkonium chloride, a quaternary ammonium compound, is one of the main ingredients in alcohol-free hand sanitizer. The positively charged nitrogen atom in the quaternary ammonium compound interacts with the phosphate group of the phospholipids in the microbial cell membrane, leading to the destabilization of the cell membrane and eventually causing the lysis of microbial cells [7].

Besides glycerol and H_2_O_2_, other minor components present in the hand sanitizer are oxidative products of ethanol, isopropanol, and methanol. The oxidative products can be formed during the prolonged storage of alcohol-based hand sanitizer at room temperature or at an elevated temperature such as in an automobile that has been parked outside. Acetaldehyde and acetone are common products formed during the storage of hand sanitizers due to the oxidation of ethanol and isopropanol, respectively. Acetaldehyde is considered a toxic chemical for animals and humans whereas low concentrations of acetone exposure have no known health hazards. In the case of acetaldehyde, it is classified as a substance reasonably anticipated to be a human carcinogen in the 15th report on carcinogens by the US Department of Health and Human Services [8]. In humans and mammals, acetaldehyde and acetone are generated in the body as a part of the main excretion mechanism of ingested alcohols, through the oxidation of ethanol to acetaldehyde and isopropanol to acetone by the enzyme alcohol dehydrogenase (ADH) [9,10,11,12]. Acetaldehyde is further metabolized into acetate by aldehyde dehydrogenase (ALDH), whereas acetone metabolism is a more complex mechanism that involves the generation of glucose, carbon dioxide, and water [9,13]. A significant amount of acetone was detected through the excretion of CO_2_ via breath [13,14]. Abnormalities in the ALDH enzyme or its inhibition can cause high levels of acetaldehyde buildup for an extended period, which has been correlated with several diseases including liver disease [10,11]. Isopropanol intoxication is a common health problem, and it is partially due to the slow metabolism of isopropanol [15,16]. Acetone is a central nervous system (CNS) depressant and has a longer elimination half-life than ethanol [14]. Exposure to acetone at low levels is not associated with any health issues. However, acute or long-term exposure to acetone can lead to health problems, including skin irritation and respiratory issues. Acetone is found in several household items including disinfectants, cleaners, and nail polish removers.

Methanol is a toxic chemical found in several household items. It is a common contaminant in ethanol due to imperfect distillation or fermentation, which led to multiple accidental methanol poisonings through alcoholic beverages [17,18]. Apart from CNS depression and metabolic acidosis, vision impairment is a characteristic symptom of methanol poisoning [19]. Methanol is metabolized into formaldehyde and formic acid by ALDH and ADH, respectively.

The accurate concentration determination of alcohols and their oxidative products is very critical in forensics, toxicology, environmental science, and several other fields [20,21,22]. Gas chromatography (GC), infrared spectroscopy (IR), and Raman spectroscopy techniques are very popular in forensics and environmental fields to determine trace amounts of chemicals [23,24,25]. Analytical sensors based on the enzymatic reaction of alcohol dehydrogenase are also used for the quantification of alcohol in biological samples [25,26,27]. Among all these methods, GC-based methods are the most reliable and robust to detect alcohols and other volatile organic compounds [28,29]. In most of the applications, GC is either coupled with a flame ionization (GC-FID) or mass spectrometry (GC/MS) detector. In certain cases, a combination of both detectors was employed [30]. Alcohol and its metabolites in blood and other complex samples have been measured widely using GC-FID and GC/MS methods [22,30,31,32,33,34]. Although the sample can be introduced into the GC system via a liquid injection system, the headspace (HS) gaseous injection method is the popular choice for alcohols and their metabolites because of their volatile nature [29]. Since the headspace sample injection system only injects the volatile components of the sample mixture, the unwanted materials, such as salts and non-volatile chemicals, do not enter into the GC/MS part of the instrument. The significance of headspace GC/MS in testing the accurate concentration of alcohol in alcohol-based hand sanitizers lies in providing specific and quantitative measures of alcohol levels, ensuring product quality, efficacy, and regulatory compliance. A brief list of studies that determined the concentration of alcohols and their related compounds with their limit of detection (LOD) and limit of quantification (LOQ) is provided in Table 1.

In this study, a new GC/MS method is developed to determine the accurate concentration of alcohols and their metabolites using deuterium-labeled analytes. The use of isotopes as an internal standard (ISTD) not only enables the unambiguous identification of peaks within complex mixtures but also facilitates the accurate determination of the analyte concentration [35]. The GC/MS method has been successfully applied in the analysis of hand sanitizer samples to determine the presence of alcohols, acetaldehyde, and acetone. The primary objective of this study was to measure levels of the oxidation products of alcohol, acetaldehyde, acetone, and methanol. The effect of temperature and storage duration of hand sanitizer was also a focus of this study.

**Table 1:** The limit of detection (LOD) and limit of quantification (LOQ) of alcohols, acetaldehyde, and acetone selected in this study, along with the reported values from previous studies.

	LOD	LOQ	Technique	Sample Types Studied	Reference
Acetaldehyde	0.5 μg/L	N/A	HS-GC/MS (NCI)	Drinking water	[28]
	N/A	104 μg/mL	HS-GC/MS	Hand sanitizers	[29]
	0.1 μg/mL	0.5 μg/mL	HS-GC/MS	Blood	[20]
	0.209 μg/mL	0.314 μg/mL	HS-GC/MS	Hand sanitizers	This study
Methanol	9 ppm	31 ppm	HS-GC/MS	Hand sanitizers	[36]
	N/A	105 μg/mL	HS-GC/MS	Hand sanitizers	[29]
	0.2 μg/mL	1 μg/mL	HS-GC/MS	Blood	[20]
	0.03 mg/mL	0.05 mg/mL	HS-GC/MS	Blood	[32]
	0.211 μg/mL	0.316 μg/mL	HS-GC/MS	Hand sanitizers	This study
Ethanol	28 nM	N/A	HS-GC/MS	Aqueous environmental	[21]
	99 μg/mL	N/A	HS-GC-FID	Blood	[22]
	0.4 μg/mL	39.5 μg/mL	HS-GC/MS	Blood	[33]
	N/A	104 μg/mL	HS-GC/MS	Hand sanitizers	[29]
	0.1 μg/mL	0.5 μg/mL	HS-GC/MS	Blood	[20]
	0.158 μg/mL	0.210 μg/mL	HS-GC/MS	Hand sanitizers	This study
Isopropanol	N/A	104 ug/mL	HS-GC/MS	Hand sanitizers	[29]
	11 μg/mL	27 μg/mL	HS-GC-FID	Medicinal drugs	[37]
	0.1 μg/mL	2 μg/mL	HS-GC/MS	Warfarin derivatives	[38]
	7 ppm	21 ppm	HS-GC/MS	Terpenes	[39]
	0.157 μg/mL	0.209 μg/mL	HS-GC/MS	Hand sanitizers	This study
Acetone	0.1 μg/mL	0.5 μg/mL	HS-GC/MS	Blood	[20]
	0.1 ppm	N/A	Sensor/IR laser	Breath	[40]
	3 μg/mL	7 μg/mL	HS-GC-FID	Medicinal drugs	[37]
	5 ppm	16 ppm	HS-GC/MS	Terpenes	[39]
	0.157 μg/mL	0.209 μg/mL	HS-GC/MS	Hand sanitizers	This study
n-Propanol	100 ppm	N/A	Sensor/CuO	CuO fiber	[41]
	100 ppb	N/A	Sensor/AgCrO_2_	Nanoparticles	[42]
	0.0100 μg/mL	0.0201 μg/mL	HS-GC/MS	Hand sanitizers	This study

## 2. Materials and Methods

### 2.1. Chemicals

Commercially available methanol, ethanol, isopropanol, n-propanol, and acetone were obtained from Fisher Scientific (Hampton, NH, USA) and acetaldehyde was obtained from Thermo Scientific (Waltham, MA, USA). Heavy isotopes were obtained from the following sources: methanol-d4, ethanol-d6, acetone-d6 (Cambridge Isotopes, Tewksbury, MA, USA), isopropanol-d8 (Thermo Chemicals, Waltham, MA, USA), and acetaldehyde-d4 (Fisher Scientific, Hampton, NH, USA).

### 2.2. Headspace Gas Chromatography/Mass Spectrometry (HS-GC/MS) Analysis

GC/MS analysis was conducted using an Agilent 8860 GC attached to a 5977B MSD and 7697A headspace sampler. The GC column used for the analysis was an Agilent J&W DB-WAX ultra inert (30 m × 0.25 mm × 0.50 μm) column. An Agilent ultra inert split liner was used in the GC inlet for the split injection (50:1). The samples were injected from a 20 mL GC headspace vial with a Teflon/silica crimp cap vial using the headspace autosampler. The headspace oven temperature, loop temperature, and transfer line temperatures were kept at 60 °C, 80 °C, and 100 °C, respectively. The sample vial was equilibrated in the headspace oven at 60 °C for 10 min prior to the injection and the sample was injected into the GC column over a period of 30 sec. The temperature of the GC inlet was kept at 120 °C. The GC oven temperature program was started with a 2 min hold at 32 °C, then increased up to 110 °C at a rate of 10 °C/min, and kept at 110 °C for 5 min. Helium was used as a carrier gas with a flow rate of 52.4 mL/min.

**Figure 1:**
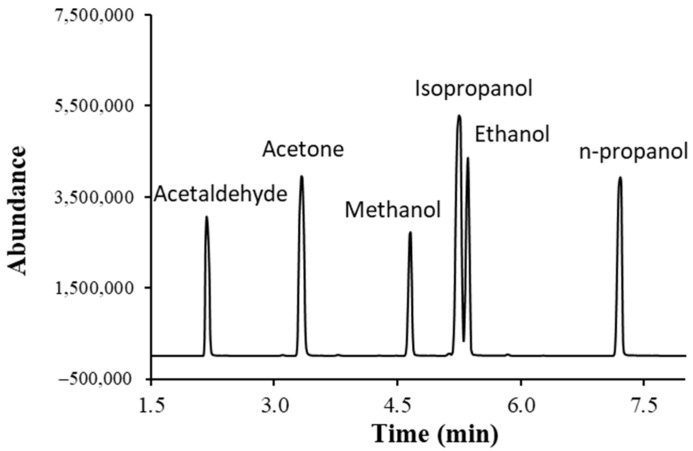
GC/MS total ion chromatogram (TIC) of a mixture of standard alcohols, acetone, and acetaldehyde.

The mass spectrometer was operated in the electron impact (EI) mode at an ionization voltage of 70 eV. The MSD transfer line temperature and source temperature were set at 250 °C and 230 °C, respectively. The selected ion monitoring (SIM) method was used for the quantification of the analytes. In the SIM method, each analyte was monitored at the following time region of the run based on the scan mode GC/MS chromatogram obtained for the analytes (Figure 1): Acetaldehyde (1.8–3.0 min), acetone (3.0–4.15 min), methanol (4.15–4.9 min), ethanol and isopropanol (4.9–7.0 min), and n-propanol (7.0–8.0 min). More details about the MS analysis are provided in Section 3.1.

### 2.3. Hand Sanitizer Sample Preparation

Commonly available gel-based hand sanitizers were purchased from local stores. Seven out of nine of them were ethanol-based hand sanitizers. Other types of samples used were isopropanol-based and alcohol-free (benzalkonium chloride) hand sanitizers.

In ethanol-based hand sanitizers, the concentration of ethanol is significantly higher than the concentration of the other analytes. Therefore, two types of sample preparation were performed, one to quantify the ethanol content and the second to quantify all the other analytes except ethanol. First, the sample for ethanol quantification was prepared by using 1.0 mL of 1000-times-diluted hand sanitizer (prepared by mixing 10 μL of undiluted hand sanitizer and 9.990 mL of dI water). Second, the sample for the quantification of other analytes (minor components) was prepared using 1 mL of the undiluted hand sanitizer.

A similar approach was used in the preparation of isopropanol-based hand sanitizer: one for the quantification of isopropanol (1.0 mL of 1000-times-diluted hand sanitizer) and the second (1.0 mL of undiluted hand sanitizer) for the quantification of other analytes. In the case of non-alcohol-based hand sanitizer, the samples were prepared by only using 1 mL of the undiluted hand sanitizer.

A known amount of the deuterium-labeled compounds of the analytes, 5 μL of methanol-d4, 10 μL of ethanol-d6, 5 μL of isopropanol-d8, 5 μL of acetaldehyde-d4, and 5 μL of acetone-d6 were also added as internal standards to each of the above samples. The concentration of the internal standards was in the range of 0.041 mg/mL to 0.087 mg/mL. All the samples were prepared in a 20 mL crimp-capped headspace GC glass vial.

For temperature-dependent studies, the samples were sealed in GC vials after preparation and kept in a water bath at different temperatures for 24 h. Similarly, for time-dependent studies, the samples were placed in a 25 °C water bath for various durations of time.

## 3. Results and Discussion

### 3.1. GC/MS Analysis of Pure Alcohols, Acetone, and Acetaldehyde

Commercially available alcohols, acetaldehyde, and acetone were used to optimize the separation of analytes and their analysis conditions. Each compound was injected individually and as a mixture to confirm the chromatographic retention time and their mass spectrometric parameters. The GC/MS chromatogram obtained after the injection of a mixture of alcohols, acetone, and acetaldehyde is provided in Figure 1. Acetaldehyde eluted first, while n-propanol eluted last. Isopropanol and ethanol eluted closely around 5.2 min.

Initially, the mass spectrometry analysis was conducted in scan mode to identify the major characteristic ions (*m*/*z*) of each analyte. Similarly, isotopes (methanol-d4, ethanol-d6, acetone-d6, isopropanol-8, and acetaldehyde-4) of the above were also injected to identify the major ions in each compound. The characteristic ions of each compound found in the scan mode analysis were used for the quantification of analytes in hand sanitizers using the selected ion monitoring (SIM) method. For each compound, there was a qualifier ion and a quantifier ion. The quantifier ions used for the SIM mode analysis were as follows: methanol (*m*/*z*: 31, 33), ethanol (*m*/*z*: 31, 33), isopropanol (*m*/*z*: 45, 49), acetaldehyde (*m*/*z*: 44, 48), and acetone (*m*/*z*: 43, 46), where the lower *m*/*z* values are from the analyte and the higher *m*/*z* is from the isotope analog of the analyte. The dwell time for each ion was set at 100 ms. The quantification of the analyte was conducted by comparing the peak area of the analyte with that of the corresponding deuterated internal standard. A detailed list of major ions and fragment ions of each analyte is provided in the Supplementary Materials.

### 3.2. Method Validation

The method developed here was validated based on specificity, accuracy, precision, linearity, limit of detection (LOD), and limit of quantification (LOQ). Each analyte was injected in triplicate at a minimum of three different concentrations. The retention time of the analyte and the area of the corresponding peaks were analyzed.

*Specificity:* The chromatographic separation and characteristic mass to charge (*m*/*z*) peaks of each analyte were monitored to check if any other interfering peak was present at the retention time of the analyte by injecting both blank and standard samples. Each compound was injected individually and as a mixture to confirm the chromatographic retention time and their mass spectrometric parameters. The analytes used in this study had enough chromatographic separation, and no interfering peak was observed. The GC/MS chromatogram obtained after the injection of a mixture of alcohols, acetone, and acetaldehyde is provided in Figure 1.

*Precision and accuracy:* The precision of the method was tested by preparing three sample replicates of standard solutions of each analyte and determining their concentrations using the method described above. The concentration value obtained for each analyte from the triplicate samples exhibited a high degree of consistency. The relative standard deviation percentages obtained for each analyte are methanol: 1.96%, ethanol: 1.64%, isopropanol: 1.97%, acetaldehyde: 1.95%, and acetone: 2.60%.

The accuracy was tested by injecting triplicate samples at concentrations of 0.001582 mg/mL, 0.001578 mg/mL, 0.00157 mg/mL, 0.00314 mg/mL, and 0.000627 mg/mL, for methanol, ethanol, isopropanol, acetaldehyde, and acetone, respectively. The observed concentration values (96–104%) were within the acceptance level.

**Figure 2:**
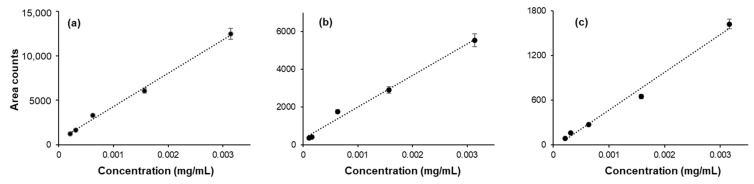
Standard curve of (**a**) acetaldehyde, (**b**) acetone, and (**c**) methanol. Standard solutions of each analyte at various concentrations were prepared and injected into HS-GC/MS. The area under the peak of each compound was plotted against the concentration of the analyte, and a linear fit was used to draw the trend line.

*Linearity, limit of detection (LOD), and limit of quantification (LOQ):* The GC/MS signal response to the concentration of analyte was checked for each analyte by preparing a standard curve. The samples for the standard curve were prepared by adding a specified amount of the analyte (concentration range: 0.000001–1.0 mg/mL) in a 20 mL glass vial. The total volume of the sample was adjusted to 1 mL by adding dI water. The standard curves obtained for acetaldehyde, acetone, and methanol are provided in Figure 2. Similar standard curves were obtained for ethanol, isopropanol, and n-propanol. The limits of detection and limits of quantification were also determined. A wide range of LOD and LOQ values are reported in the literature. The LOD and LOQ values determined in this work, along with those from a few selected previous studies, are provided in Table 1.

### 3.3. GC/MS Analysis of Hand Sanitizers Kept in Common Storage Conditions and in Parked Cars

Most of the hand sanitizers selected in this study contain ethanol as the main ingredient. The concentration of alcohols, acetaldehyde, and acetone present in each hand sanitizer was determined using the internal standard method described above. The ethanol-based hand sanitizers, HS1, HS2, HS3, HS4, and HS7, were stored at room temperature, whereas the HS6 and HS8 sanitizers were stored in a parked car outside. The isopropanol-based hand sanitizer, HS9, and the non-alcohol-based hand sanitizer, HS5, were stored at room temperature.

#### 3.3.1. Analysis of Major Components in Hand Sanitizer

In all the ethanol-based hand sanitizers, the ethanol concentration was found to be in the range of 520–560 mg/mL, which is in agreement with the expected values for ethanol-based hand sanitizers [4,43]. The amount of ethanol observed in the room-temperature samples was higher than that observed in the parked car samples. This is due to the high temperature inside the car during the parked stage. At elevated temperatures, ethanol evaporates from the hand sanitizers leading to a decrease in ethanol concentration in the parked car. In the isopropanol-based hand sanitizer (HS9), the major component was found to be isopropanol (540 mg/mL). In the alcohol-free hand sanitizer (HS5), no detectable amount of ethanol or other alcohol was determined. The main ingredient present in the HS5 sanitizer was benzalkonium chloride. No attempt was made to determine the concentration of benzalkonium chloride using GC/MS.

#### 3.3.2. Quantification of Minor Components in Hand Sanitizer

The alcohol-related minor components found in the ethanol-based hand sanitizers were methanol, isopropanol, acetaldehyde, and n-propanol (Table 2). The highest concentration of methanol observed was 0.0152 mg/mL, whereas the lowest concentration was 0.00014 mg/mL, which was observed in the HS7 and HS5 samples, respectively. These levels of methanol were lower than the FDA-approved level of 0.63 mg/mL [44]. Methanol is a common impurity found in hand sanitizers as well as in other consumer products [36,43,45]. Isopropanol was found as a minor component in all ethanol-based hand sanitizers included in this study. The highest amount of isopropanol (13.84 mg/mL) was observed in the HS2 hand sanitizer. Isopropanol was not detected in the HS5 hand sanitizer. In most of the hand sanitizers, a small amount of n-propanol (0.0009–0.15 mg/mL) was also observed, except in the HS1 and HS5 hand sanitizers. Here also, the amount of n-propanol observed was lower than the approved FDA level, which is 1.0 mg/mL. In all ethanol-based hand sanitizers, acetaldehyde was observed as a minor component. This is consistent with previous studies [43]. The highest amount of acetaldehyde was observed in the HS4 sample, which was stored at room temperature for a long time (2 months). Hand sanitizer samples from the parked cars (HS6 and HS8) had the lowest amount (0.001–0.002 mg/mL) of acetaldehyde. This could be due to the low boiling point of acetaldehyde, which must have evaporated out of the hand sanitizer bottle since it was equipped with a push-down pump dispenser. In the case of acetone, a detectable amount was not found in ethanol or benzalkylchloride-based hand sanitizers. However, a significant level of acetone was found in isopropanol-based hand sanitizers.

### 3.4. Effect of Storage Time on Acetaldehyde Formation in Ethanol-Based Hand Sanitizers

In all ethanol-based hand sanitizers, a small amount of acetaldehyde was observed. In the HS4 and HS7 sanitizers, significantly higher amounts of acetaldehyde were observed compared with other ethanol-based hand sanitizers. The lowest amount of acetaldehyde was found in the HS5 sample, the alcohol-free hand sanitizer. Acetaldehyde is formed by the oxidation of ethanol at room temperature. Previous studies have shown that acetaldehyde can be formed in alcoholic beverages and other consumer products containing ethanol due to its long storage time [43,46,47]. Acetaldehyde is a reactive compound, which can undergo reactions with other compounds as well. For example, in alcoholic beverages, especially wine, it reacts with flavonoids and produces other chemicals [48].

**Figure 3:**
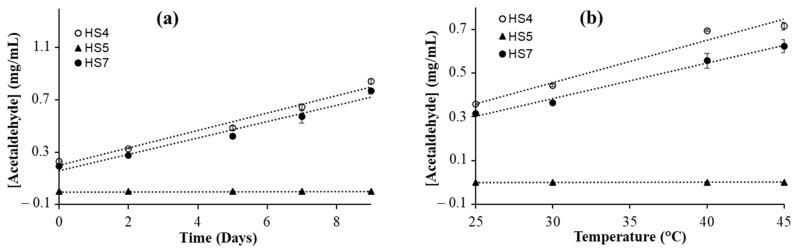
Acetaldehyde formation as a function of time and temperature. Acetaldehyde concentration in hand sanitizers (**a**) after keeping the hand sanitizer at room temperature for various durations of time and (**b**) after keeping the hand sanitizer at various temperatures for 24 h. The HS4 and HS7 samples are ethanol-based hand sanitizers, whereas the HS5 sample is an alcohol-free hand sanitizer.

To determine the effect of storage time on the oxidation of alcohol, the hand sanitizer samples were kept in a sealed GC/MS vial for various durations of time at room temperature (25 °C) and the amount of acetaldehyde formed in each sample was analyzed. It was observed that the concentration of acetaldehyde increased linearly with time (Figure 3a). The concentration of acetaldehyde in the HS4 and HS7 sanitizers increased by more than 200% over a period of seven days. In the HS5 sample, an alcohol-free hand sanitizer, no formation of acetaldehyde was observed. The HS5 hand sanitizer was used as a control for this study. These results agree with the expected effect of storage time because acetaldehyde is formed from ethanol oxidation. The significant increase in acetaldehyde concentration in hand sanitizers may result from the presence of hydrogen peroxide, a common additive in hand sanitizers [3,4]. Hydrogen peroxide is a powerful oxidizing agent commonly used in various chemical reactions [49]. The optimal condition for H_2_O_2_-mediated oxidation is a slightly acidic environment in the presence of transition metal ions. It was reported earlier that the oxidation of softwood kraft pulp using H_2_O_2_ converts hydroxyl groups in carbohydrates to carbonyl groups, a reaction similar to the conversion of alcohols to acetaldehyde and acetone [50]. The prolonged storage of hand sanitizer that contains a significant amount of H_2_O_2_ poses a higher chance of producing more acetaldehyde and can be a potential risk. Chemical oxidation of ethanol typically does not occur without the presence of oxidizing agents. This may explain why ethanol oxidation is minimal in alcoholic beverages since they may not contain a significant concentration of H_2_O_2_. Also, some alcoholic beverages (e.g., wine) contain antioxidants [51].

### 3.5. Effect of Temperature on Acetaldehyde Formation in Ethanol-Based Hand Sanitizers

Keeping hand sanitizers in cars and in other high-temperature places is a very common practice in daily life. The hand sanitizers HS6 and HS8 were kept in a car for two weeks before the analyses. As we can see in Table 2, acetaldehyde concentration was low in those samples compared with other samples. The bottles of the HS6 and HS8 sanitizers were equipped with a push-pump system to deliver the hand sanitizer. Since it was not completely sealed, ethanol and acetaldehyde must have evaporated from the bottle. The ethanol concentration was also lower in these samples compared with the other samples stored at room temperature.

To check the effect of temperature on acetaldehyde formation, the hand sanitizer samples were prepared in sealed GC vials for each temperature individually and kept at different temperatures for 24 h. The highest temperature used was 45 °C, which is very close to the inside temperature of a parked car in the summer. For this experiment, two ethanol-based hand sanitizers (HS4 and HS7) and a non-alcohol-based hand sanitizer (HS5) were used. A significant increase in the concentration of acetaldehyde was observed in both ethanol-based hand sanitizers (Figure 3b). However, no such increase was observed in the HS5 sanitizer, which was expected. In both ethanol-based hand sanitizers, we observed a linear dependence on acetaldehyde formation with temperature. An increase of more than 100% in acetaldehyde concentration was observed in the HS4 and HS7 sanitizers when the samples were kept at 45 °C for 24 h, compared with the samples kept at 25 °C for the same duration. This is not very surprising because temperature typically increases the rate of most reactions. Previous kinetic studies on the ethanol oxidation reaction, involving the reaction between alpha hydroxyethyl radical and oxygen, showed an increased production of acetaldehyde at higher temperatures [52]. This is consistent with the results obtained here. As described earlier, H_2_O_2_ plays a role here as well. The efficacy studies of ethanol-based hand sanitizer against microbes have shown that their efficiency is optimal at 22 °C and decreases when the temperature is above 40 °C [53], which could be due to the decrease in ethanol concentration via evaporation as well as the conversion to acetaldehyde.

### 3.6. Effect of Time and Temperature on Acetone Formation in Isopropanol-Based Hand Sanitizer

**Figure 4:**
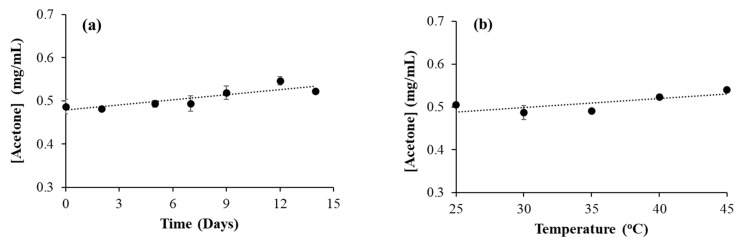
Acetone formation as a function of time and temperature. Acetone concentration in hand sanitizers (**a**) after keeping the hand sanitizer at room temperature for various durations of time and (**b**) after keeping the hand sanitizer at various temperatures for 24 h.

Similar to ethanol, isopropanol can also undergo oxidation to form its oxidation product, acetone. In the isopropanol-based hand sanitizer HS9, a small amount of acetone was observed (Table 2). In order to observe the effect of time on oxidation, a time-dependent experiment was conducted with the HS9 samples at 30 °C. The samples were kept in a GC/MS vial, sealed, and placed in a 30 °C water bath for various time durations. However, no significant increase in acetone concentration was observed during this time (Figure 4a). In seven days, the increase in acetone concentration was less than 10%. Similarly, the effect of temperature on acetone formation was also checked by heating the HS9 sample at various temperatures for 24 h. Here also, there was no significant increase in acetone concentration observed (Figure 4b).

The above results are different from what we observed with acetaldehyde formation in ethanol-based hand sanitizers. Acetaldehyde concentration increased with both time and temperature, whereas acetone concentration did not change significantly with time or temperature. This can be due to the difference in the nature of the OH functional groups in ethanol and isopropanol. The OH functional group in ethanol is a primary alcohol, whereas in isopropanol it is a secondary alcohol. The oxidation of secondary alcohol is always more difficult than that of primary alcohol. It has also been previously reported that, in enzymatic reactions, secondary alcohols oxidize more slowly than primary alcohols [54].

## 4. Conclusions

The headspace GC/MS method developed in this study proves to be a reliable and sensitive method to quantify trace amounts of impurities such as acetaldehyde, acetone, methanol, and other alcohols present in hand sanitizer and similar samples. The incorporation of heavy-labeled isotopes assists in definitively identifying the target compound within a complex mixture. This method has a broad application in various sample types including body fluids and other complex mixtures, making it suitable for diverse fields such as forensics and toxicology. The oxidative products of alcohol formed in hand sanitizers (e.g., acetaldehyde) are toxic chemicals that can be formed very easily at room temperature conditions. An increase of 10–20 °C above room temperature, which is analogous to the temperature inside a parked car, can significantly elevate the generation of acetaldehyde in ethanol-based hand sanitizers. If the hand sanitizer bottle has a permanent opening, such as a push-down pump dispenser bottle, it can release acetaldehyde vapors into the atmosphere inside the car, exposing passengers to it. Similarly, prolonged storage of hand sanitizer at room temperature in closed containers can result in the accumulation of high concentrations of acetaldehyde inside the bottle. The presence of H_2_O_2_ in hand sanitizers can enhance the generation of acetaldehyde. The amount of H_2_O_2_ can vary based on the manufacturers and can have a significant impact on the efficiency of hand sanitizer. The structural differences between ethanol and isopropanol influence their extent of oxidation. Since isopropanol-based hand sanitizers have a slower oxidation rate than ethanol-based hand sanitizers, isopropanol-based hand sanitizers can be more efficient than ethanol-based hand sanitizers when they need to be stored for longer periods or exposed to higher temperatures.

## Figures and Tables

**Table 2:** Minor components found in hand sanitizers and their quantities in the selected samples.

Sample	Major Component	Minor Components (mg/mL)				
		Methanol	Isopropanol	n-Propanol	Acetaldehyde	Acetone
HS1	Ethanol	0.000921	0.4539	n.d	0.00585	n.d
HS2	Ethanol	0.000312	13.840	0.00267	0.00402	n.d
HS3	Ethanol	0.00139	0.4967	0.000907	0.05206	n.d
HS4	Ethanol	0.00389	0.4969	0.000474	0.23120	n.d
HS5	BenzalkCl	0.000143	n.d	n.d	0.00062	n.d
HS6	Ethanol	0.00457	0.5035	0.00143	0.00228	n.d
HS7	Ethanol	0.01519	0.6050	0.1520	0.19420	n.d
HS8	Ethanol	0.00204	0.5059	0.00829	0.00158	n.d
HS9	Isopropanol	0.00156	N/A	0.02492	0.02043	0.6966
FDA limit		NMT 0.63	NR	NMT 1.00	NMT 0.05	NMT 4.40

FDA sample refers to the individual limit of the impurities required by the FDA for hand sanitizer samples [44]. NMT: no more than; NR: not required, and n.d: not detected.

## Data Availability

The data presented in this study are stored with the corresponding author and are available upon request. Additional data are available as Supplementary Materials.

## Supplementary Materials

The following supporting information can be downloaded at: https://www.mdpi.com/1660-4601/21/1/74/s1, Table S1: GC/MS mass spectrometric ions and major fragment ions of alcohols, acetaldehyde, and acetone.
