# Supplementary material for: Determination and Quantification of Acetaldehyde, Acetone, and Methanol in Hand Sanitizers Using Headspace GC/MS: Effect of Storage Time and Temperature
Source: Int J Environ Res Public Health. 2024 Jan 9;21(1):74. doi: 10.3390/ijerph21010074 (PMC10815429; doi:10.3390/ijerph21010074)
Supplement: Supplementary file 1 [file ijerph-21-00074-s001.zip › ijerph-2728183-supplementary.pdf]

Supplementary Information

Determination and quantification of acetaldehyde, acetone and methanol in Hand sanitizes using Headspace GC-MS: Effect of storage time and temperature.

Chessa To and Jacob A. Theruvathu*

Table S1: GC-MS analysis of active ingredients and impurities possibly found in commercial hand sanitizers.

Compound	Retention time (min)	Maor ion (m/z)	Other ions (m/z)
**Methanol**	4.66	31	32
**Methanol-d4**	4.581	33	34, 35
**Ethanol**	5.358	31	45, 46, 43
**Ethanol-d6**	5.246	33	49, 51, 34
**Isopropanol**	5.262	45	43, 41, 39
**Isopropanol-d8**	5.132	49	46, 50
**N-propanol**	7.220	31	59, 42, 60
**Acetaldehyde**	2.197	44	43, 42, 45
**Acetaldehyde-d4**	2.197	48	46, 44

*Main ion fragments account for 100% in relative abundance. Other ion fragments were arranged in descending relative abundance.
